# Increased Risk of Dementia in Patients With Erectile Dysfunction: A Population-Based, Propensity Score-Matched, Longitudinal Follow-Up Study

**DOI:** 10.1097/MD.0000000000000990

**Published:** 2015-06-19

**Authors:** Chun-Ming Yang, Yuan-Chi Shen, Shih-Feng Weng, Jhi-Joung Wang, Kai-Jen Tien

**Affiliations:** From the Department of Neurology, Chi Mei Medical Center, Tainan (C-MY); Department of Urology, Kaohsiung Chang Gung Memorial Hospital (Y-CS); Cheng Shiu University, Kaohsiung (Y-CS); Department of Medical Research, Chi Mei Medical Center (S-FW, J-JW); Department of Hospital and Health Care Administration, Chia Nan University of Pharmacy and Science (S-FW); Division of Endocrinology and Metabolism, Department of Internal Medicine, Chi Mei Medical Center (K-JT); and Department of Senior Citizen Service Management, Chia Nan University of Pharmacy and Science, Tainan, Taiwan (K-JT).

## Abstract

Erectile dysfunction (ED) is a well-known predictor for future cardiovascular and cerebrovascular disease. However, the relationship between ED and dementia has rarely been examined. This study investigates the longitudinal risk for Alzheimer's disease and non-Alzheimer dementia in patients with ED.

We collected a random sample of 1,000,000 individuals from Taiwan's National Health Insurance database. From this sample, we identified 4153 patients with newly diagnosed ED between 2000 and 2009 and compared them with a matched cohort of 20,765 patients without ED. All patients were tracked for 7 years from the index date to identify which of them subsequently developed dementia.

During the 7-year follow-up period, the incidence rate of dementia in the ED cohort was 35.33 per 10,000 person-years. In the comparison groups, it was 21.67 per 10,000 person-years. After adjustment for patients characteristics and comorbidities, patients with ED were 1.68-times more likely to develop dementia than patients without ED (95% CI = 1.34–2.10, *P* < 0.0001). In addition, older patients and those with diabetes, hypertension, chronic kidney disease, stroke, depression, and anxiety were found to be at increased risk for dementia. Analyzing the data by dementia type, we found the hazard risk for Alzheimer's disease and non-Alzheimer dementia to be greater in patients with ED (adjusted HR 1.68, 95% CI = 1.31–2.16, *P* < 0.0001 and 1.63, 95% CI = 1.02–2.62, *P* = 0.0429, respectively). Log-rank test revealed that patients with ED had significantly higher cumulative incidence rates of dementia than those without (*P* < 0.0001).

Patients with ED are at an increased risk for dementia later in life.

## INTRODUCTION

Erectile dysfunction (ED), which is the inability to develop or maintain penile erection during sexual intercourse, is a complex and heterogeneous disorder possibly related to endocrinological, vasculogenic, psychological, and neurogenic diseases.^1^ It often clusters with hypertension, diabetes mellitus, hyperlipidemia, and metabolic syndrome and has been reported to be an early predictor of macrovascular disease.^2,3^ One meta-analysis of cohort studies reported that ED is associated with a 48% increase in risk of cardiovascular (CV) disease (CVD), 46% increase in risk of coronary heart disease, 35% increase in risk of stroke, and a 19% increase in risk of all-cause mortality.^4^ Another meta-analysis of cohort studies enrolling 92,757 subjects also reported an association between ED and increased risk of future CV events and mortality, especially in young subjects and subjects with intermediate baseline CV risk.^5^ The risk conferred by ED on such events is comparable to that of the traditional CV risk factors, and thus meticulous CVD investigation is recommended for ED patients.^4,5^ ED was also reported to be associated with neurological disorders, including migraine, epilepsy, and Parkinson's disease.^6–8^ It has been proposed that some potential pathologic changes such as endothelial dysfunction, insulin resistance, increased inflammatory mediators, increased sympathetic activity, and dysfunctional nitric oxide (NO) pathway may explain the association between ED and macrovascular disease.^1,9^ Moreover, as suggested by artery size hypothesis in Montorsi et al, atherosclerosis affects all major vascular beds to the same extent and it affects small caliber arteries, such as the penile artery, often earlier to large caliber arteries by an equally sized plaque.^10^

Dementia, a serious loss of global cognitive ability in a previously healthy person, can be categorized into different subtypes according to cause. Alzheimer's disease, a neurodegenerative disorder, accounts for about 50% of all cases of dementia.^11^ The pathological hallmarks of Alzheimer's disease are amyloid plaque and neurofibrillary tangles that damage the structure and function of neurons and synapses.^12^ It has been proposed that potential risk genes, systemic inflammation, immunologic response, and insulin resistance contribute to the development of Alzheimer's disease.^13,14^ Many medical conditions are also associated with the risk of Alzheimer's disease, including obesity, stroke, diabetes, midlife hypertension, and hypercholesterolemia.^12^ Vascular dementia accounts for 20–25% of all cases of dementia and comprises the majority of cases of non-Alzheimer dementia. Vascular dementia is caused by cerebrovascular disease that impairs blood flow to the brain. It may be prevented to some extent by controlling risk factors such as diabetes, hypertension, smoking, and dyslipidemia.^11^

Although ED is often seen as a harbinger of CVD, few studies investigate the relationship between ED and neurodegenerative diseases, such as dementia. One cross-sectional study by Dourado et al showed that ED is common among individuals with dementia and suggested that it is related to cognitive decline.^15^ Another cross-sectional study of 651 men reported an association between ED and poorer cognitive performance, particularly attention–executive–psychomotor speed tasks. These findings underscore the importance of further study of the possibility that ED can serve as a predictor of cognitive health.^16^ Cross-sectional studies, however, cannot by design confer directionality on observed associations. Without the use of longitudinal study designs, it is unclear whether the presence of ED may be antecedent to the development of dementia and whether ED is associated with an increased risk of dementia later in life. While both ED and dementia are associated with CV risk factors, we hypothesize there should be an association between ED and dementia. To find out, we used a national population-based cohort in Taiwan to investigate the risk for the development of dementia in patients with ED and without ED over a 7-year period.

## METHODS

### Data Sources

The Taiwan National Health Insurance (NHI) Program is a universal healthcare system that covers 98% (in 2009) of the country's population of 23.3 million, making it one of world's largest and most complete population-based datasets. The NHI Research Database (NHIRD) provides all the claim data of the healthcare services, including hospitalization and outpatient clinics, and has been collected and encrypted by patient's identification number. Our data source is a randomly sampled cohort (in 2000) of 1 million people (about 4% of the enrollees) from the general population in NHIRD. The database contained all 1996–2011 claims data. The sex, age, and health care costs were not different between the sample group and all enrollees at that time. The Institutional Review Board of Chi Mei Medical Center approved the protocol of this study. Informed consent was not needed because of no identifiable personal information in the dataset.

### Study Sample

A retrospective cohort study was conducted using 2 study groups, a newly diagnosed ED group and a matched non-ED (control) group, recruited between 2000 and 2009. Patients were defined as having ED if they had at least 2 outpatient service claims with a diagnosis of ED (ICD-9-CM code 607.84) at any hospital or local medical clinic or if they had a single hospitalization in which ED (ICD-9-CM code 607.84) was found in 1 of the 5 spaces used to report their diagnoses when hospitalized. Index date was the date of first ED diagnosis. Patients diagnosed as having ED before 2000 were excluded. We further classified dementia into Alzheimer's disease and non-Alzheimer dementia. Patients diagnosed with Alzheimer's disease (ICD9-CM code: 290.0, 290.1, 290.2, 290.3, 294.1, 331.0) or non-Alzheimer dementia (ICD9-CM code: 046.1, 290.4, 331.1, 331.2, 331.7, 331.8, 331.9) before the ED diagnosis were also excluded. For the accuracy of dementia diagnosis, patients had to have had at least 2 outpatient service claims or 1 inpatient service claim of dementia. In addition, to be included, patients had to have received neuropsychological tests such as clinical dementia rating (CDR; order code: 45052), mini-mental status examination (MMSE; order code: 45046), or cognitive ability screening instrument (CASI; order code 45058). They also had to have undergone brain imaging such as brain CT (order code: 33067, 33068, 33069, 33070) or MRI (order code: 33084, 33085).

### Control Group

Controls (non-ED group), 5 patients for each ED patient, were randomly selected from the same dataset. These controls did not have an ED diagnosis and were matched by age, residence area, income, and comorbid diseases including diabetes mellitus, hypertension, coronary heart disease, hyperlipidemia, stroke, chronic kidney disease, depression, anxiety, and thyroid disorder by propensity score matching. Propensity score matching reduced selection bias in our hypothesis because it can bundle many confounding covariates that may be present in an observational study with this number of variables. Controls diagnosed with Alzheimer's disease or non-Alzheimer dementia before the ED diagnosis were also excluded.

Demographic data, including age, geographic area of residence, and monthly income in NTD, were recorded. The baseline comorbidity data we collected were diabetes mellitus (ICD9CM code: 250), hypertension (ICD9CM code: 401–405), coronary heart disease (ICD9CM code: 410–414), hyperlipidemia (ICD9CM code: 242), stroke (430–438) and chronic kidney disease (ICD9CM code: 582, 583, 585, 586, 588), depression (ICD9CM code: 311, 296.2, 296.3, 300.4), anxiety (ICD9CM code: 300.0–300.9 (but not 300.4)), and thyroid disorder (ICD9CM code: 240–246) because these disease entities are known to be critical factors affecting the risk of dementia. These comorbid conditions were included if they were diagnosed during hospitalization or in 3 or more ambulatory care claims coded 1 year before the index medical care date. Follow-up time in person-years (PY) was calculated for each person until dementia was diagnosed, death, or the end of 2011.

### Statistical Analyses

Pearson's *χ*^2^ tests were used to estimate the differences in age group, sex, comorbid medical disorders, and socio-demographic data between the 2 cohorts. The dementia incidence rate (per 100,00 person years) was calculated as the number of dementia patients during the follow-up period divided by the total person-years for each group. A Poisson regression was used to estimate the incidence rate ratio (IRR) in comparing the risk of dementia in ED group and the control group. Adjusted HRs for developing dementia between patients with and without ED were obtained by Cox proportional hazard regression analysis after adjusting for possible confounding factors (diabetes, hypertension, coronary heart disease, chronic kidney disease, hyperlipidemia, stroke, depression, anxiety, thyroid disorder, geographic area, and monthly income). Kaplan–Meier curves and the log-rank test were used to analyze the proportion of dementia patients and to compare risk difference between 2 cohorts. A 2-sided *P* value <0.05 was considered significant. All statistical operations were performed using the SAS 9.3.1 Statistical Package (SAS Institute, Inc, Cary, NC.

## RESULTS

As can be seen in Table 1, a summary of the baseline characteristics and comorbid conditions of the 2 groups, 4153 ED patients and 20,765 matched non-ED patients, were followed for 7 years. Underlying comorbidities, residence area, and income were also matched in the present study.

**TABLE 1:**
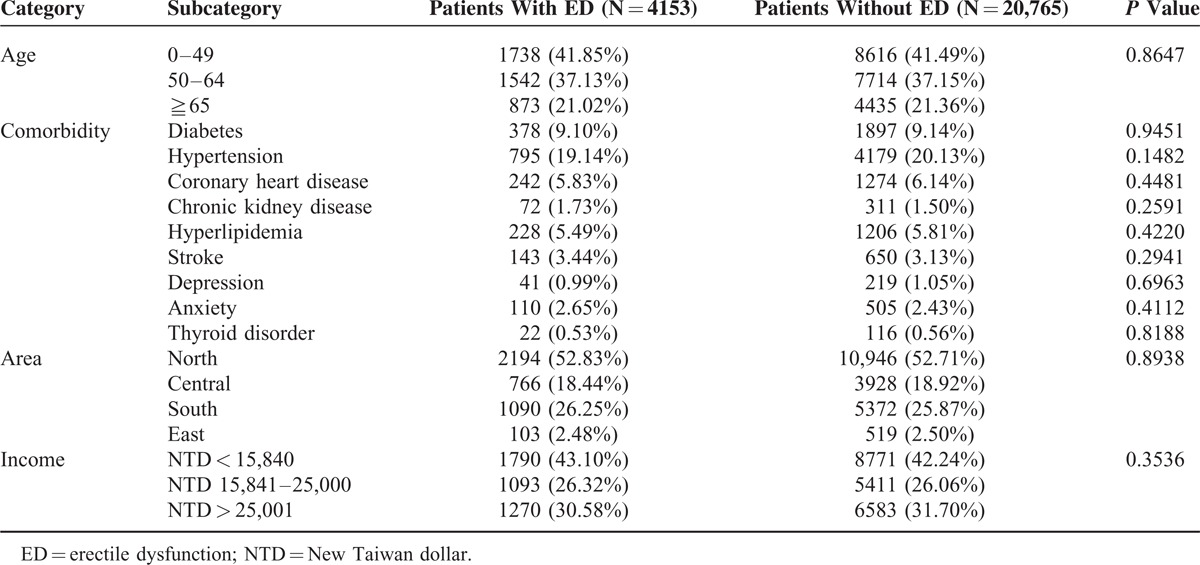
Demographic Characteristics and Comorbid Medical Disorders for Patients With and Without Erectile Dysfunction in Taiwan

Patients with ED had significantly higher incidence of dementia than the controls (Table 2). Of the 4153 ED patients, 105 (2.5%) were diagnosed as having dementia during the 7-year follow-up (35.33 per 10,000 person-years). Of the 20,765 controls, 317 (1.5%) developed dementia (21.67 per 10,000 person-years). The incidence rate ratio (IRR) for dementia was 1.63 (95% CI 1.31–2.03; *P* < 0.0001) for patients with ED versus non-ED. After adjusting for age, DM, HTN, CHD, CKD, hyperlipidemia, stroke, depression, anxiety, thyroid disorder, geographic area, and income, ED patients were found to be 1.68 times more likely to develop dementia than the controls (adjusted HR = 1.68, 95% CI = 1.34–2.10, *P* < 0.0001). In addition, elderly patients (age ≥65) were at 27 times greater risk of dementia than the younger ones (age <50). Patients with DM, HTN, CKD, stroke, depression, and anxiety were also predisposed to dementia, their respective adjusted HRs being 1.48, 1.29, 1.47, 1.43, 3.53, and 1.59.

**TABLE 2:**
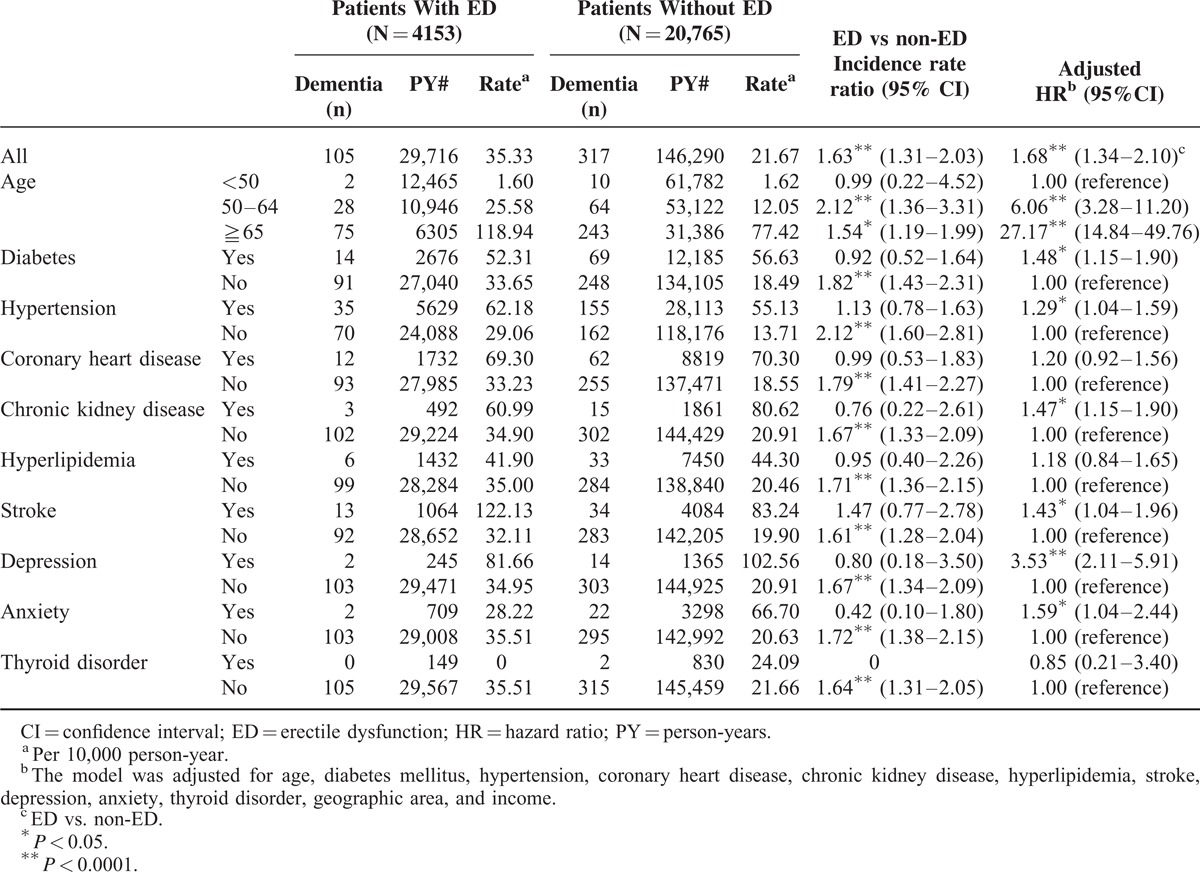
Risk for Dementia (Alzheimer's Disease Plus Non-Alzheimer Dementia) in Patients With and Without Erectile Dysfunction

**FIGURE 1:**
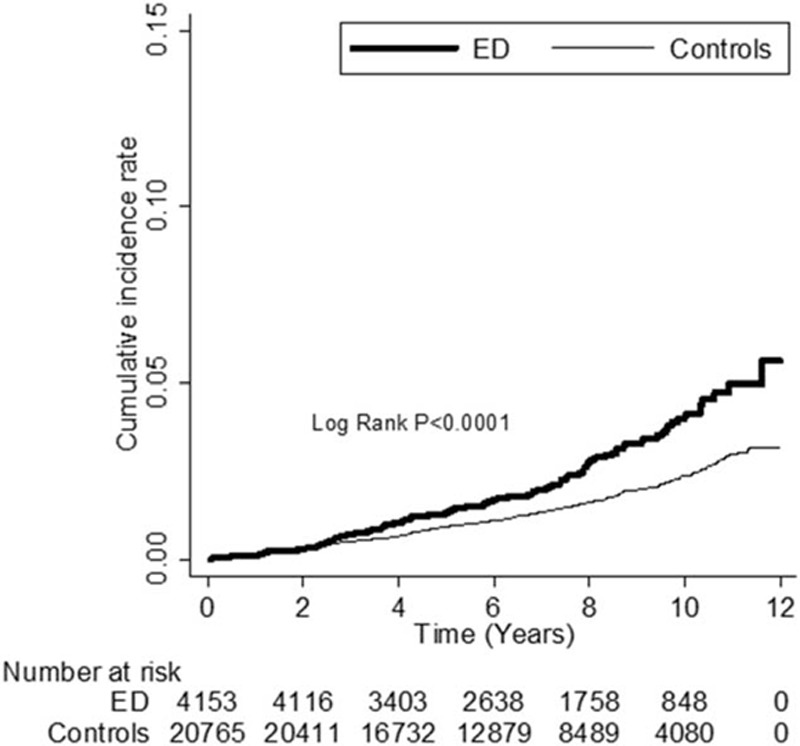
The cumulative incidence rate for dementia for patients with erectile dysfunction (ED) and without ED (log-rank *P* value < 0.0001).

**FIGURE 2:**
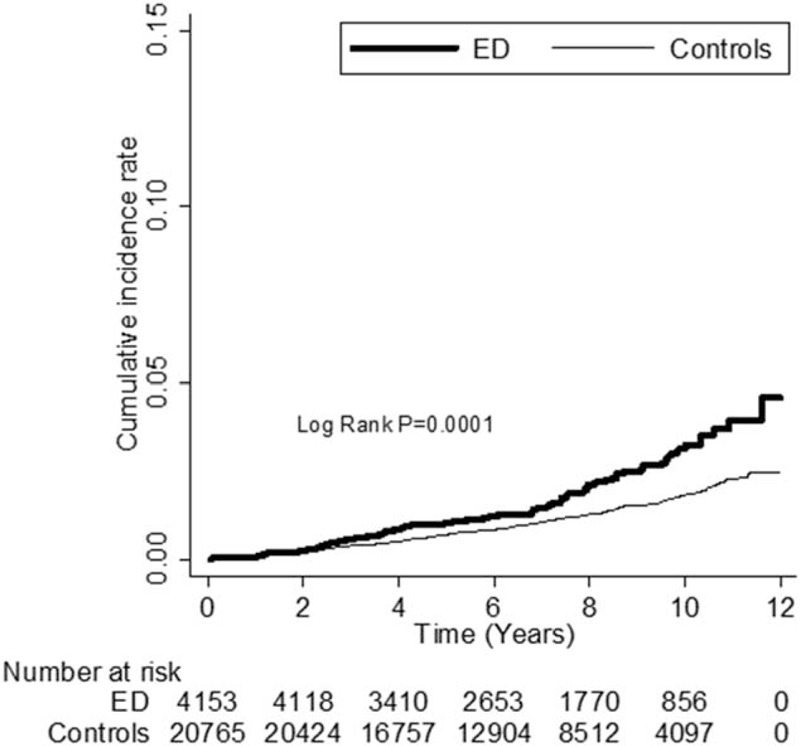
The cumulative incidence rate for Alzheimer's disease for patients with erectile dysfunction (ED) and without ED (log-rank *P* value = 0.0001).

**FIGURE 3:**
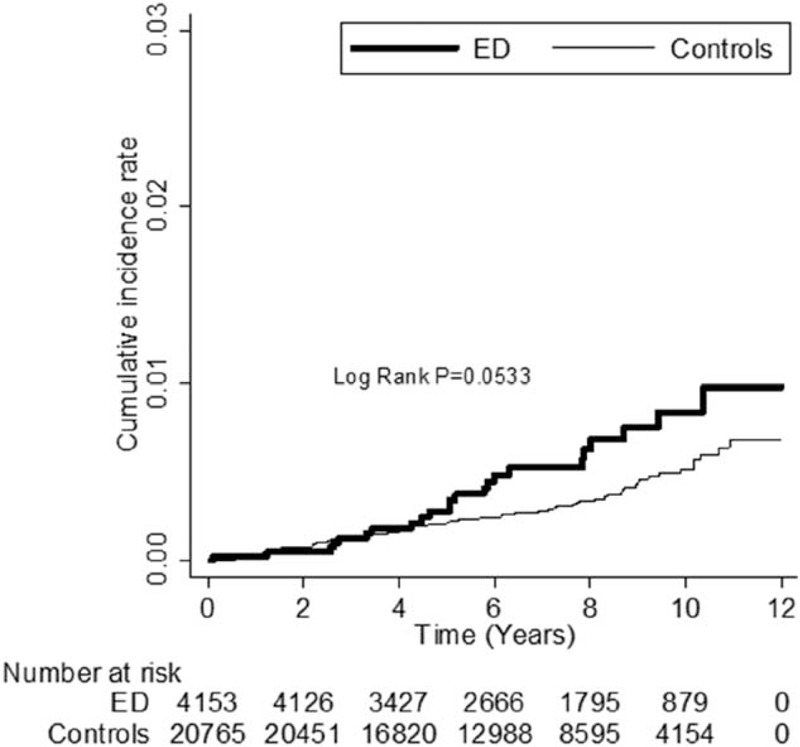
The cumulative incidence rate for non-Alzheimer dementia for patients with erectile dysfunction (ED) and without ED (log-rank *P* value = 0.0533).

As can be seen in Table 3, an analysis by dementia subgroup, patients with ED were at higher risk of developing Alzheimer's disease and non-Alzheimer dementia than the controls (adjusted HRs 1.68, 95% CI = 1.31–2.16, *P* < 0.0001 and 1.63, 95% CI = 1.02–2.62, *P* = 0.0429, respectively). Kaplan–Meier analysis revealed that, compared to the controls, patients with ED had significantly higher incidence of dementia (log-rank test *P* < 0.0001) (Figure 1), Alzheimer's disease (log-rank test *P* = 0.0001) (Figure 2), and borderline higher incidence of non-Alzheimer dementia (log-rank test *P* = 0.0533) (Figure 3).

**Table 3:**
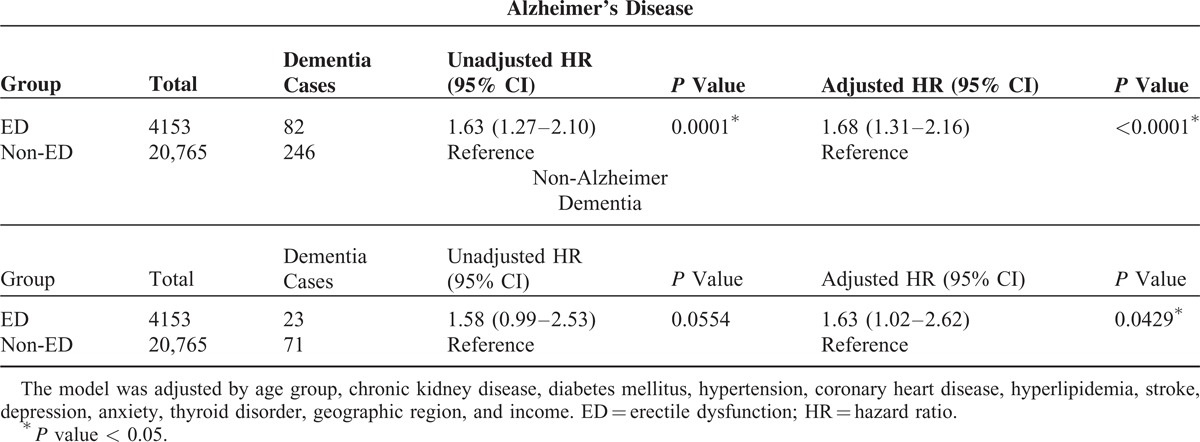
Risk for Alzheimer's Disease and Non-Alzheimer Dementia Among Patients With and Without Erectile Dysfunction

## DISCUSSION

To the best of our knowledge, this is the first nationwide population-based study to investigate the risk of dementia among patients with ED in an Asian population. We found incidence rate for dementia in patients with ED to be 35.33 per 10,000 person-years and to be 1.68-times higher than the comparison groups during the 7-year follow-up period, after adjusting for age, medical comorbidities, geographic area, and monthly income. Risks of Alzheimer's disease and non-Alzheimer dementia were both greater in the ED group (adjusted HRs 1.68 and 1.63, respectively).

Although cross-sectional studies have reported ED to be common among individuals with Alzheimer's disease,^15,17^ those studies generally considered ED to be increased secondary to cognitive decline and dementia. Very few previous reports have investigated whether patients with ED are at increased risk of cognitive impairment later in life. Moore and the colleagues, conducting a cross-sectional study to evaluate the cognitive difference between men with ED and normal control,^16^ reported that men with ED had poorer cognitive performance even after adjusting for CV risk factors. They suggested that ED was an indicator for early compromised cognition and should be seen as a predictor for cognitive health. Postuma et al, investigating the relationship between autonomic dysfunction and dementia with Lewy bodies,^18^ measured various autonomic parameters and found that systolic blood pressure drop, ED, and constipation predicted dementia as long as 5 years before a dementia diagnosis.

Although the contributing mechanism underlying the association between ED and dementia is likely complex, there are some possible explanations. First, patients with ED have lower bioavailable and free testosterone than those without ED.^19^ Androgen might act as a neuroprotector against neurodegenerative disease.^20^ A 3-year follow-up study by Nagai et al, investigating the association between testosterone level and the cognition as assessed by mini-mental state examination (MMSE), found blood-free testosterone level to be an independent predictor of decrease in cognition.^21^ Low testosterone level can result in faster declines in cognitive function, as androgen has been implicated in lower levels of beta-amyloid protein, which may be important because the accumulation of the protein is widely considered a key initiating factor in the development of Alzheimer's disease.^22,23^ Therefore, testosterone depletion might raise the risk of Alzheimer's disease. Second, ED has been highly associated with insulin resistance and may be considered a very early clinical sign of developing insulin resistance.^9,24^ Insulin resistance might exacerbate neurodegenerative process in Alzheimer's disease^25^ and has been highly associated with a decline in cognition and dementia.^26^ Third, although the cause of ED is multifactorial in nature, it is typically associated with endothelial dysfunction and oxidative stress.^27,28^ Endothelial dysfunction can disturb the beta-amyloid homeostasis leading to neuronal injury in the brain parenchyma.^29^ Oxidative stress has been reported by many studies to possibly play a key pathological role in Alzheimer's disease.^30–32^ Fourth, traditional CV risk factors such as diabetes, hypertension, and dyslipidemia are believed to predict the incidence of Alzheimer's disease and dementia.^33–35^ Since ED often clusters with metabolic comorbidities and CV risk factors, it is not surprising in our study that a diagnosis ED would raise the risk for dementia. The two share many similar risk factors. The present study controlled for underlying comorbidities, including vascular, neurological, psychological, and endocrinological, and metabolic disorders and found that hazard risk for dementia was still significant in the ED group. The association between ED and dementia may be, in part, due to these comorbidities, but they do not explain the entire relationship between ED and dementia.

This study has some limitations. First, the coding of ED and dementia were taken from diagnoses listed in the NHI database. Thus, some coding errors should be considered. Patients were identified as having ED and dementia only if they had been diagnosed at least 2 times during the study period to enhance diagnostic validity. To maximize case ascertainment, only the patients meeting the above criteria and receiving neuropsychological tests (MMSE, CASI, or CDR) as well as brain imaging (CT or MRI) were diagnosed as having dementia. In Taiwan, the physicians use the International Index of Erectile Dysfunction (IIEF-5) questionnaire to quantify the symptoms and assess the severity of ED. Because the original data of the IIEF-5 and neuropsychological tests were not recorded in the database, we could not evaluate the severity of both diseases. Second, ED is a taboo subject and patients are reluctant to discuss sex in Taiwan. Thus, there may be some cases of ED which were not identified in the control group. Even under this circumstance, the risk was still clearly demonstrated. The true risk may be even greater if we can definitely verify the ED and control groups. Second, individual information, including environmental factors, education level, marital status, diet, smoking, and alcohol use, were not available. Single men are supposed to have higher risk of depression which may affect both the erectile function and the cognitive level. Nonetheless, some factors related to the unavailable personal information would, at least in part, be reflected in the presentation of comorbidities, such as depression, anxiety, and income status, which were covered in our analysis. Laboratory data such as nitric oxide (NO) level were also not available in the database. Impaired NO bioactivity is one of the major pathogenic mechanisms of ED and may also contribute to neurodegenerative disorders such as Alzheimer's disease.^36,37^ Although we attempted to overcome the problem of selection bias by using a propensity score matching strategy, it can only be addressed using observable variables. Unobservable variables may still confound our results. Further study incorporating finer clinical details is needed to clarify the effects of these factors. Despite these limitations, the strength of this investigation lies in the fact that it studied a large-scale population covering nearly all of the residents in Taiwan and it had a longitudinal design, thus minimizing referral and selection biases.

In conclusion, this study found increased risk of Alzheimer's disease and non-Alzheimer dementia among patients with ED. ED might be seen as an early predictor of dementia. Physicians should be alerted to this association so that they may identify these patients earlier. Further studies are needed to gather more information to explore the mechanisms underlying these relationships.
